# Glyceraldehyde-3-Phosphate Dehydrogenase of *Babesia microti* Is a Plasminogen- and Actin-Binding Protein

**DOI:** 10.3389/fvets.2019.00228

**Published:** 2019-07-09

**Authors:** Xiangye Liu, Huiqin Li, Hongkuan Deng, Chen Zheng, Hongru Yan, Zetian Chen, Anning Bian, Jiaxu Chen, Kuiyang Zheng

**Affiliations:** ^1^Jiangsu Key Laboratory of Immunity and Metabolism, Department of Pathogenic Biology and Immunology, Xuzhou Medical University, Xuzhou, China; ^2^School of Life Sciences, Shandong University of Technology, Zibo, China; ^3^Key Laboratory of Parasite and Vector Biology, Chinese Center for Disease Control and Prevention, WHO Collaborating Centre for Malaria, Schistosomiasis and Filariasis, Ministry of Health of China, National Institute of Parasitic Diseases, Shanghai, China

**Keywords:** *Babesia microti*, glyceraldehyde-3-phosphate dehydrogenase, plasminogen, α-actin, binding protein

## Abstract

*Babesia microti*, an intraerythrocytic protozoa, can cause an emerging tick-borne disease—Human babesiosis. The parasite can successfully invade host red blood cells owing to the assistance of molecules expressed by babesia. Glyceraldehyde-3-phosphate dehydrogenase (GAPDH), the housekeeping intracellular glycolytic enzyme, can also be expressed in the external of cells, where contributes to binding to several molecules such as plasminogen and actin. In the present study, we identified *B. microti* GAPDH (BmGAPDH) and generated the recombinant BmGAPDH (rBmGAPDH) via an *E. coli* expression system. Furthermore, we confirmed its catalytic dehydration activity *in vitro*. Moreover, we also demonstrated that rBmGAPDH could bind to human plasminogen and mouse α-actin. In addition, we demonstrated that rBmGAPDH could recognize anti-*B. microti* mouse serum. In conclusion, BmGAPDH is a multifunctional glycolytic enzyme, which can bind to host plasminogen and α-actin.

## Introduction

*Babesia micorti*, an intraerythrocytic apicomplexan protozoa, is the primary causative pathogen of human babesiosis, which is an emerging tick-borne diseases in the worldwide (1). Generally, three classical transmission pathways play the key role in the process of babesia infection, including tick biting, blood transfusions, and trans-placental transmission (1). Red blood cells (RBCs), the initially invaded mammalian host cell, can be damaged accompanied with the development and multiplication of the parasite, of which leads to non-specific symptoms of the patients such as elevated liver enzyme levels, thrombocytopenia, and hemolytic anemia (2).

As reported, the parasite can successfully invade host RBCs owing to the assistance of molecules expressed by babesia (3). Recently, numerous numbers of parasite molecules have been identified from genome projects or functional RBC-binding assays to facilitate babesia successful dissemination and invasion within the host (3, 4). Therefore, well-understood the invasion mechanism of the parasite will benefit to exploit the functional molecules, which can be potentially used to diagnose, therapy, and prevent human babesiosis.

Glycolysis is an ancient metabolic pathway, being found across kingdoms and in most organisms, which is catalyzed by 10 enzymes (5). As known, glycolysis has also been considered as the main energy resources of apicomplexan parasites during their erythrocytic stages (6). Interestingly, apicomplexan glycolytic enzymes lack special extracellular targeting motifs, but can be presented at the cell surface of apicomplexan parasites (7). A number of such enzymes, which play key metabolic roles in glycolysis, also perform new and non-catalytic roles that usually interacted with interaction to host molecules, *e.g.*, enolase (8).

A well-known housekeeping enzyme and one of the key glycolytic enzymes, glyceraldehyde-3-phosphate dehydrogenase (GAPDH, EC: 1.2.1.12), is able to catalyze the conversion of glyceraldehyde-3-phosphate (G3P) to 1-3 di-phosphoglycerate (1,3-DPG) at the sixth step of glycolysis (9). It has been demonstrated that GAPDH possesses multiply functions in eukaryotic pathogens, which are involved in gene expression controlling, cell signaling, and interaction with other proteins (10). Recent studies also suggest that GAPDH is an important virulence factor, plays a pivotal role during the infection process of numerous pathogens, which may be a potential vaccine candidate for protection against pathogenic diseases (10, 11). A single GAPDH gene has been identified on chromosome I of *B. microti* (6). However, the role of GAPDH has not been identified during *B. microti* infection processes.

In the present study, we identified the protein structure of *B. microti* GAPDH (BmGAPDH), and then generated the recombinant BmGAPDH (rBmGAPDH) through an *E. coli* expression system. Subsequently, we demonstrated that the rBmGAPDH catalyzed G3P dehydration to 1,3-DPG. In addition, we also revealed that rBmGAPDH could bind to human plasminogen and mouse α-actin.

## Materials and Methods

### Animals and Parasites

Female Balb/c mice (4–6 weeks old) were purchased from Beijing Vital River Laboratory Animal Technology Co., Ltd (Beijing, China) and bred under specific-pathogen-Free (SPF) conditions at the Animal Center of Xuzhou Medical University. Our study was performed in accordance with the recommendations of the Guide for Care and Use of Laboratory Animals of the Laboratory Animal Ethics Committee of Xuzhou Medical University. The Laboratory Animal Ethics Committee of Xuzhou Medical University approved all our animal experimental protocols (Permit Number: 201547).

The *Babesia microti* strain (ATCC® PRA-99^TM^) was obtained from the National Institute of Parasitic Diseases, Chinese Center for Disease Control and Prevention (Shanghai, China) and maintained in our laboratory. The procedure for mouse infection of *B. microti* was performed as our previous description (8). In brief, female Balb/c mice were intraperitoneally inoculated with *B. microti* infected blood, and Giemsa-staining was used to identify the percentage of parasitized red blood cells (RBCs).

### BmGAPDH Characterization

BmGAPDH amino acid sequences were obtained with BLAST searches against the NCBI protein database, which derived from the published genomic sequences of *B. microti* (6). The signature sequences and specific domains were identified with online software of ScanProsite (http://prosite.expasy.org/scanprosite), and MotifScan (http://myhits.isb-sib.ch/cgi-bin/motif_scan), Multiple protein sequences were aligned and analyzed with online software of MUSCLE multiple sequence alignment tool (http://www.ebi.ac.uk/Tools/msa/clustalo.)

In order to characterize the interactions of BmGAPDH with plasminogen and actin, the structure of BmGAPDH, human plasminogen (Accession number: AAA60113) and mouse α-actin (Accession number: AAA37164) were modeled with SWISS MODEL (https://swissmodel.expasy.org). Then docking assays between BmGAPDH and plasminogen or α-actin were performed with Cluspro 2.0 to identify the interaction and binding sites. Finally, the best model was selected and visualized by PyMOL from 30 models of each assay.

### Recombinant BmGAPDH Preparation

The open reading frame (ORF) of BmGAPDH was synthesized at GENEWIZ® (GENEWIZ Suzhou, China). *EcoR*I and *Nde*I restriction enzyme sites were, respectively, added to BmGAPDH 5' and 3' ends to easily clone into the expression vector—pET-28a (+), then the recombinant plasmid was transformed into *Escherichia coli* BL21 star (DE3) competent cells. The *E. coli* were induced with 0.4 mM isopropyl-β-D-thiogalactopyranoside (IPTG) for 18 h at 25°C, then they were lysed with a sonicator; finally, the recombinant protein was purified on an HiTrap column (GE Healthcare, PA, USA). Following sodium dodecyl sulfate polyacrylamide gel electrophoresis (SDS-PAGE), the purified protein was analyzed by using coomassie brilliant blue staining. Following quantitated with a BCA protein assay kit (Beyotime Biotechnology, Shanghai, China), the recombinant BmGAPDH (rBmGAPDH) was divided into aliquots and stored at −80°C before used.

### Generation of Mouse Polyclonal Antibody Against rBmGAPDH

Mouse polyclonal antibodies against rBmGAPDH were prepared as our previous description (8). In brief, 40-μg purified rBmGAPDH mixed with MONTANIDE^TM^ ISA 70 VG adjuvant (Seppic, Puteaux, France) was subcutaneously injected into 4–6 weeks old female Balb/c mice every 2 weeks. The mice serum was collected at 1 week following the third immunization. Finally, the titer and specificity of antibody was, respectively, evaluated by enzyme-linked immunosorbent assay (ELISA) and Western-blot.

### Immunoprecipitation and Pull-Down Assay

Immunoprecipitation assay was performed as preciously described (8). In brief, 10 μl of anti-rBmGAPDH antibodies was co-incubated with 1 mg of *B. microti* infected or normal mouse RBC lysates overnight at 4°C, then mixed with 30 μl of A/G Sepharose protein slurry for 2 h at 4°C. Following three washes with PBS, the precipitate was analyzed by using SDS-PAGE and Western-blot. For pull-down assay, 2 μg of rBmGAPDH protein was co-incubated with 2 mg of normal mouse RBC lysates overnight at 4°C. The next day, 20 μl of Ni Sepharose was mixed and incubated overnight at 4°C. Following three washes with PBS, the precipitate was analyzed with SDS-PAGE and Western-blot.

### rBmGAPDH Enzymatic Activity Measurement

The enzymatic activity of rBmGAPDH was identified by measuring of absorbance enhancement at 340 nm following previous description (12). In brief, the reaction was performed in a 200 μl reaction buffer (50 mM Glycine, 50 mM potassium dihydrogen phosphate, and 5 mM EDTA Na_2_, pH 9.5) with 0.5 mM NAD^+^ (Sigma–Aldrich, MO, USA) and 0.2 mM G3P, (Sigma–Aldrich, MO, USA) and with different concentrations of purified rBmGAPDH (6, 12, 40, and 50 ng/μl) at 25°C. BSA was used as a negative control. rBmGAPDH activity was determined using a reaction buffer system covering a pH range of 5.5–11.5, and temperature range of 16–60°C. Reactions were initiated by the addition of rBmGAPDH (2.4 μg/reaction) and continuous readings were carried out every 10 min for 120 min. Finally, the values of Michaelis–Menten constant (*K*_m_) and maximum velocity (*V*_max_) for the glycolysis substrate G3P was calculated using computerized non-linear regression analysis of the data fitted to the Michaelis–Menten equation with Graphpad Prism 5.0 (GraphPad Software, Inc., USA).

### Plasminogen-Binding Assay

rBmGAPDH binding to plasminogen (PLG) was detected by using ELISA following previous description (8). Briefly, 4 μg of human PLG (Sigma–Aldrich, MO, USA) was coated in each well of 96-well plate; following blocked with 2% BSA in PBS, 100 μl/well rBmGAPDH solution with different concentrations of purified rBmGAPDH (0, 0.4, 0.8, and 1.2 ng/μl) was incubated for 2 h at 37°C. Following three washes, wells were, respectively, incubated with primary antibody—polyclonal mouse anti-rBmGAPDH serum (1:200 dilution) and secondary antibody—HRP-conjugated anti-mouse IgG (1:10000 dilution) for 1 h at 37°C. Finally, the absorbance was read at 450 nm and each assay was performed in triplicate.

### Statistical Analysis

All data were repeated at least three times and presented as mean ± standard deviation (SD). Statistical analysis was performed with one-way analysis of variance (ANOVA) and Tukey's multiple comparison tests by using Graphpad Prism 5.0 (GraphPad Software, Inc. USA) and *p* < 0.05 was considered as significant difference.

## Results

### *B. microti* Glyceraldehyde-3-Phosphate Dehydrogenase (BmGAPDH)

**Figure 1:**
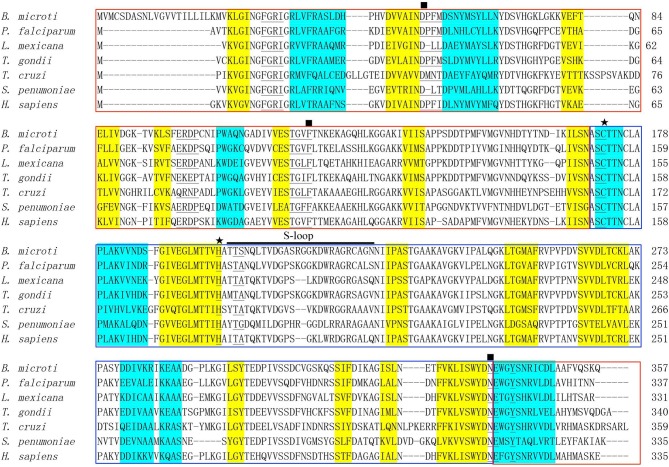
Bioinformatics analysis of BmGAPDH. Multiple sequence alignment of GAPDHs from *Babesia microti* (XP_012647129), *Plasmodium falciparum* (AAK30144), *Leishmania mexicana* (CAA46323), *Toxoplasma gondii* (AAK20420), *Trypanosoma cruzi* (CAA37080), *Streptococcus cerevisiae* (CAD44376), and *Homo sapiens* (NP_002037) was performed by using the ClustalX program. The alpha helix and beta sheet secondary structures are, respectively, shaded in yellow and turquoise. The NAD^+^ binding domains is boxed with red whereas the catalytic domain is boxed with blue. The conserved NAD^+^ binding and glyceraldehyde-3-phosphate (G3P) interacting residues are, respectively, marked by rectangles and stars. The residues of NAD^+^ binding pocket are marked with underlines. S-loop is indicated with horizontal black line.

An online search for *B. microti* proteomic sequences identified a protein (accession number: XP_012647129) containing 357 amino acids, which is encoded by 1,074 nucleotides (6). The molecular weight of BmGAPDH was predicted as 38.3 kDa and its PI value was 6.98. ScanProsite search results showed that a GAPDH signature motif spanning residues 170–177 (^170^ASCTTNCL^177^) presented in the protein. A representative alignment of GAPDHs revealed the functional NAD^+^ binding domain (residues 1–169 and 339–357) and the catalytic domain (residues 170–338) (Figure 1). Classical Rossmann-folded α/β domain is the NAD^+^ binding domain of BmGAPDH, and three NAD^+^ binding residues including Asp^54^, Phe^121^, and Asn^338^ were conserved. Moreover, the NAD^+^ binding pocket, which mostly consisted of residues of ^30^Phe-Gly-Arg-Ile^33^, ^54^Asp-Pro-Phe-Met^57^, ^98^Glu-Arg-Asp-Pro^101^, ^118^Thr-Gly-Cys-Phe^121^, ^171^Ser-Cys^172^, ^202^Thr-Ser^203^, ^337^Asp-Asn^338^, and Tyr^342^, was conserved. Interestingly, the conserved residues Cys^172^ and His^199^ were involved in glyceraldehyde-3-phosphate (G3P) interacting sites. In addition, the protein presented an S-loop spanning residues 201–226 (^201^TTSNQLTVDGASRGGKDWRAGRCAGN^226^), which has been considered important for all GAPDHs (Figure 1).

**Figure 2:**
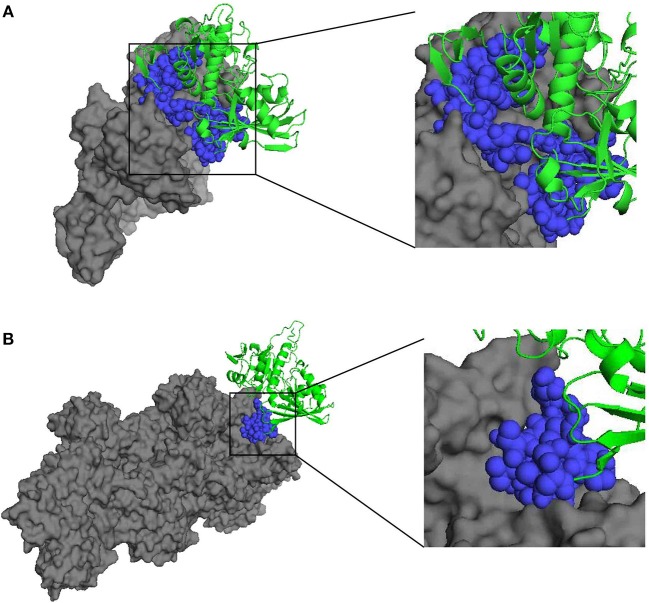
Three-dimensional structure of docking complex between BmGAPDH and human plasminogen or mouse α-actin. Molecular docking between BmGAPDH and human plasminogen or mouse α-actin was performed with ClusPro 2.0 server and visualized with PyMOL program. BmGAPDH was marked as green cartoon and human plasminogen **(A)** or mouse α-actin **(B)** was marked as gray surface, the putative binding segmentation was enlarged in right of the figure.

In addition, the docking analysis results showed that BmGAPDH could bind to both human plasminogen and mouse α-actin (Figure 2). Further analysis indicated that three predicted plasminogen-biding motifs were characterized at ^55^PFMDSNYMSYLLNYDSVHGK^74^, ^96^SFERD^100^, and ^203^SNQLTVDGASRGGKDWRAGRCAGN^226^ (Figure 2A). Moreover, a predicted α-actin-binding site was conserved at ^203^SNQLTVDGASRGGKDWRAGRCAGN^226^ (Figure 2B).

### Recombinant BmGAPDH Preparation and Antigenic Analysis

**Figure 3:**
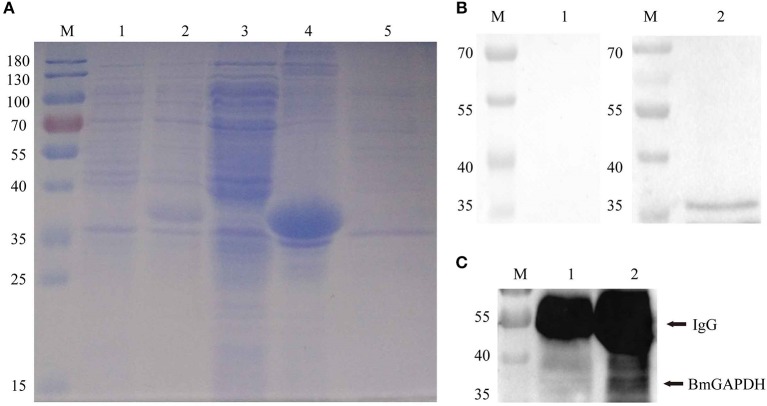
Recombinant BmGAPDH (rBmGAPDH) preparation and antigenic analysis. **(A)** Preparation of rBmGAPDH. Lane M: prestained standard protein marker; Lane 1: total cellular proteins of cell lysates; Lane 2: total cellular proteins of induced cell lysates; Lane 3: supernatant of induced cell lysates; Lane 4: sediment of induced cell lysates; Lane 5: purified rBmGAPDH protein. **(B)** Western-blot analysis of rBmGAPDH. Lane M: standard protein marker; Lane 1: purified rBmGAPDH proteins were probed with normal mouse serum; Lane 2: purified rBmGAPDH proteins were probed with *B. microti*-infected mouse serum. **(C)** immunoprecipitation analysis of the native BmGAPDH. Lane M: standard protein marker; Lane 1: normal mouse erythrocyte lysate; Lane 2: *B. microti*-infected erythrocyte lysate.

Following synthesized, the full-length cDNA of BmGAPDH was cloned into the pET-28a (+) plasmid with a His-tag domain. The rBmGAPDH protein was successfully expressed in *E. coli*, and then purified with Ni-affinity chromatography. SDS-PAGE results showed that His-tagged rBmGAPDH protein migrated at ~38 kDa, which was consistent with our predicted size (Figure 3A). Western-blot analysis results revealed that a 38 kDa protein band was recognized by *B. microti*-infected mouse serum, whereas no band was recognized with normal mouse serum (Figure 3B).

After three immunizations with purified rBmGAPDH, mouse anti-rBmGAPDH polyclonal antibodies were generated. Furthermore, the antibodies were used to identify native BmGAPDH protein in *B. microti*, which was performed with immunoprecipitation. The results showed that a 38 kDa protein band was detected in the lysates of *B. microti*-infected mouse erythrocyte, but no band was detected in the lysates of normal mouse erythrocyte (Figure 3C).

### Determination of rBmGAPDH Enzymatic Activity

**Figure 4:**
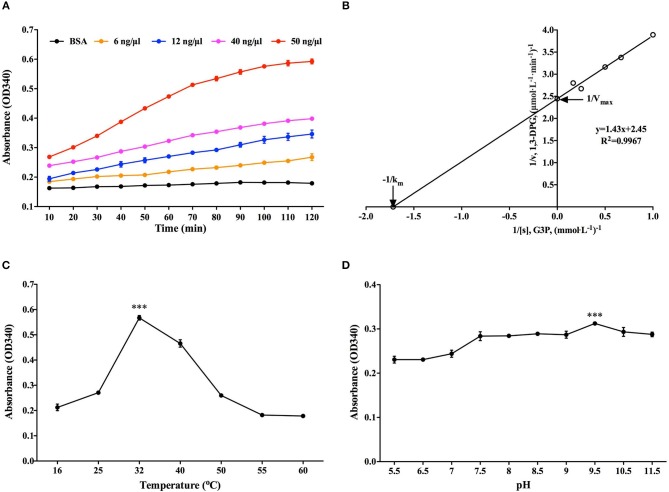
Determination of rBmGAPDH enzymatic activity. **(A)** The enzymatic activity increased with increasing concentrations of rBmGAPDH. **(B)** The Lineweaver-Burk plot was used to calculated *K*_m_ (0.583 mM) and *V*_max_ (0.401 μmol/L/min) for rBmGAPDH. **(C)** rBmGAPDH enzymatic activity was maximal at 32°C. **(D)** rBmGAPDH enzymatic activity was maximal at pH 9.5. Relative activity of rBmGAPDH was determined by OD340 absorbance. All data are represented as the mean ± SD from triplicate experiments. Significant differences are denoted by ^***^ for *p* < 0.001.

G3P reduction assay was employed to determine the classical enzymatic activity of rBmGAPDH, and BSA was used as a negative control. As shown in Figure 4A, the absorbance at 340 nm of NADPH gradually increased at 10–120 min, indicating that rBmGAPDH possesses catalytic activity. Moreover, the enzymatic activity increased following the increasing of rBmGAPDH from 6 to 50 ng/μl. The Michaelis constant (*K*_m_) and maximum velocity (*V*_max_) was, respectively, calculated as 0.583 mM and 0.401 μmol/L/min, which are the kinetics of the conversion reaction (Figure 4B). Furthermore, our results also showed that rBmGAPDH has maximal activity at 32°C and pH 9.5 (Figures 4C,D).

### Binding Activity of rBmGAPDH to Plasminogen and α-Actin

**Figure 5:**
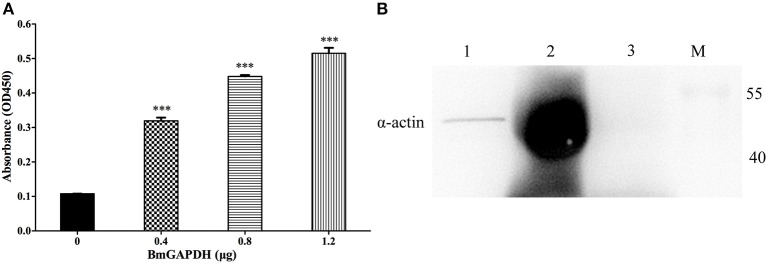
Binding activity of rBmGAPDH to plasminogen and α-actin. **(A)** Enzyme-linked immunosorbent assay (ELISA) was performed to characterize the ability of rBmGAPDH binding to human plasminogen, BSA was chosen as a negative control for non-specific binding. rBmGAPDH binding activity was determined by absorbance at OD450. All data shown represent the mean ± SD from triplicate experiments. Significant differences compared to control are denoted by ^***^p < 0.001. **(B)** Pull-down assay was performed to analyze rBmGAPDH binding to mouse α-actin. Lane M: standard protein marker; Lane 1: normal mouse erythrocyte lysate (Input); Lane 2: rBmGAPDH pull-down normal mouse erythrocyte lysate; Lane 3: negative control.

ELISA binding assays were performed to investigate rBmGAPDH's ability to bind human plasminogen. As shown in Figure 5A, the absorbance at 450 nm of rBmGAPDH groups was significantly higher than that of BSA negative control groups (*p* < 0.001). Moreover, the absorbance increased with increasing of quality of rBmGAPDH, which suggests that rBmGAPDH is able to bind to human plasminogen. In addition, GAPDH has also been reported to bind to actin, thus pull-down assays were performed to determine whether rBmGAPDH has this ability. Our results showed that a 42 kDa protein band was determined in both input and pull-down lanes, which indicated that rBmGAPDH could bind to mouse α-actin (Figure 5B).

## Discussion

Glyceraldehyde-3-phosphate dehydrogenase (GAPDH), one of the key housekeeping glycolytic enzymes, lacks special extracellular targeting motifs, but can usually present at the surface of pathogens (7). Accordingly, GAPDH has been identified possessing several other roles than its glycolysis function, including attachment to other proteins, evasion from immune surveillance of the host, and ultimately a role in microbial virulence (10). Therefore, it suggests that GAPDH may be a potential vaccine candidate for protection against pathogenic diseases.

In our present study, a 38.3 kDa protein derived from the apicomplexan parasite *B. microti*, was characterized to have a signature GAPDH domain. Furthermore, multiple sequence alignment revealed that the NAD^+^ binding sites, G3P interacting sites, and S-loop spanning residues were conserved when compared to other GAPDHs derived from *P. falciparum, L. mexicana, T. gondii, T. cruzi, S. cerevisiae*, and *H. sapiens* (13) (Figure 1). Interestingly, there is no secretory signal sequence or special targeting motifs contributing to its extracellular localization, but GAPDH can also be presented at the cell surface of parasite. However, the mechanism is not well-understood. For GAPDHs, NAD^+^ binding sites and G3P interacting sites are the classical domains, and S-loop containing the hydrophobic sequences is also considered important to form the core of proteins. Moreover, it has also been demonstrated that the plasminogen-binding motifs and α-actin-binding motifs were conserved in *B. microti* GAPDH (BmGAPDH), suggesting the potential binding ability of BmGAPDH to plasminogen and actin protein (Figure 2). Interestingly, our results indicated that both plasminogen and actin shared a same binding site, which was conserved at ^203^SNQLTVDGASRGGKDWRAGRCAGN^226^. Altogether, BmGAPDH contains NAD^+^ binding residues, catalytic residues, S-loops, plasminogen-, and actin-binding sites.

Subsequently, we generated anti-BmGAPDH mouse serum with purified recombinant BmGAPDH, which prepared in an *E. coli* BL21 (DE3) expression system. Our results showed that rBmGAPDH could be recognized by mouse serum infected with *B. microti* (Figure 3), which indicated that mouse was able to produce antibodies against BmGAPDH during *B. microti* infection. It has been reported that plasmodium GAPDH showed interesting potential as a malaria diagnostic biomarker (14). Accordingly, BmGAPDH could be a potential diagnostic biomarker for babesiosis. Interestingly, multiple sequence alignment results revealed that BmGAPDH had some homology with other GAPDH derived from *P. falciparum, L. mexicana, T. gondii, T. cruzi* (Figure 1). Therefore, the applicability of BmGAPDH in diagnosis tests should be fully verified in the future.

Usually, the same as other housekeeping proteins, GAPDH has been recognized as an important cytosolic protein. As a glycolytic enzyme, GAPDH ought to catalyze the conversion of G3P to 1,3-DPG (9). Our results indicated that the enzymatic activity of purified rBmGAPDH could be influenced by both temperature and pH values. Interestingly, rBmGAPDH could possess the highest enzymatic activity at 32°C and pH 9.5 (Figure 4). It suggests that BmGAPDH can highly adapt the changing surrounding environment. Michaelis constant (*K*_m_) is one of the best-known characters of enzyme kinetics. Here, we calculated the *K*_m_ values of BmGAPDH as 0.583 mM, which was slightly higher than that for *T. cruzi* GAPDH (0.5 mM) (Figure 4) (15). This may be due to the fact that both of the parasites are blood parasitic protozoa.

As the proenzyme of plasmin, plasminogen play a key role in fibrinolytic system (16). Moreover, plasminogen can bind to several cell surface proteins forming a complex, which plays a crucial role in both physiologic and pathologic processes such as inflammation, thrombosis, and cancer (16). In the past decades, several distinct plasminogen-binding proteins have been identified, such as α-enolase, annexin A, and Histone H2B (17). Recently, our previous studies have also demonstrated that *B. microti* enolase is able to bind to plasminogen through its lysine residues (8). Interestingly, it has been revealed that the surface localized and extracellular GAPDH is also a plasminogen binding protein in variety of pathogens (18, 19). In the present study, our ELISA experiment results also indicated that rBmGAPDH could bind to human plasminogen (Figure 5A). Moreover, GAPDH is also known to interact with actin, and facilitates actin polymerization (20). Here, our results indicated that rBmGAPDH could interact with mouse α-actin (Figure 5B). Our docking results indicated that plasminogen shared a same BmGAPDH-binding site with α-actin (Figure 2). However, different analysis procedures were performed to characterize the binding ability of BmGAPDH with plasminogen and α-actin in the present study; the possibility of competitive binding between plasminogen and α-actin should be further identified in the future.

Surprisingly, it has been reported that cell-surface actin could bind to plasminogen (17). However, the interactions among BmGAPDH, plasminogen, and actin in the process of *B. microti* infection should be issued in the future. Taken together, BmGAPDH is a multifunctional protein, which may play putative roles as virulence factors in *B. microti*.

## Conclusion

We identified a 38.3 kDa protein derived from *B. microti*, which possessed a highly conserved active site of glycolytic enzyme—GAPDH. Moreover, BmGAPDH was confirmed to catalyze the reversible dehydration of G3P to yield 1,3-DPG, and bind to plasminogen and α-actin, respectively. In addition, we demonstrated that anti-*B. microti* mouse serum could recognize BmGAPDH.

## Data Availability

The datasets used and/or analyzed in the present study are available from the corresponding authors on reasonable request.

## Author Contributions

XL conceived and designed the experiments. HL, HD, CZ, HY, ZC, and AB performed the experiments. HD, CZ, and XL analyzed the data. XL, JC, and KZ contributed reagents, materials, and analysis tools. XL, HL, and HD wrote the paper. All authors read and approved the final version of the manuscript.

### Conflict of Interest Statement

The authors declare that the research was conducted in the absence of any commercial or financial relationships that could be construed as a potential conflict of interest.
